# Supplementary material for: Limitations of MRSF-TDDFT for Applications in Photochemistry
Source: J Phys Chem Lett. 2026 Jul 23;17(31):8944–52. doi: 10.1021/acs.jpclett.6c01200 (PMC13455105; doi:10.1021/acs.jpclett.6c01200)
Supplement: Supplementary file 1 [file jz6c01200_si_001.pdf]

Supporting Information: Limitations of MRSF-TDDFT for applications in photochemistry

Jiří Janoš,*,†,‡ Andrew J. Orr-Ewing,‡ Basile F. E. Curchod,‡ and Petr Slavíček*,†

†*Department of Physical Chemistry, University of Chemistry and Technology, Technická 5, Prague 6, 166 28, Czech Republic*

‡*School of Chemistry, University of Bristol, Bristol BS8 1TS, United Kingdom*

E-mail: jiri.janos@vscht.cz; petr.slavicek@vscht.cz

Contents

1 Full characterization of the excited electronic states of naphthalene S-3

2 Analysis of the electronic configurations generated by MRSF-TDDFT with different triplet references S-4

3 The effect of enforcing orbital continuity in MRSF-TDDFT: the case of ortho-nitrophenol S-5

4 Ethyl diazoacetate results with the CAM-B3LYP functional S-7

5 Active-space orbitals used in XMS4-CASPT2(6,6) S-8

References S-9

1 Full characterization of the excited electronic states of naphthalene

While Ref. S1 reports the $1^1B^-_{3u}$, $1^1B^+_{2u}$, $1^1B^+_{3u}$ and $2^1B^+_{2u}$ states as the lowest four adiabatic excited states S_1–S_4, their calculation was not converged with respect to the number of excited states. We converged the calculation in that respect and showed that two states were missing, in agreement with Ref. S2, see Tab. S1. One of these states was also not captured by MRSF-TDDFT as it exhibits a mixed character of HOMO−1→LUMO+2 and HOMO−2→LUMO+1 transitions, further emphasizing the impact of **Limitation 1**.

Table S1: Excitation energies ΔE, oscillator strengths f, and dominant electronic configurations for selected excited electronic states of naphthalene calculated with different electronic-structure methods. The terms of the electronic states were identified from Ref. S2.

State[a]	ΔE (eV)	f	Configurations[a]
EOM-CCSD/def2-SVP			
$1^1B^-_{3u}$ (S_1)	4.51	0.0001	$0.47h_1l - 0.46hl_1$
$1^1B^+_{2u}$ (S_2)	5.37	0.0890	$0.64hl$
$2^1A^+_g$ (S_3)	6.35	0.0000	$0.45h_1l_2 + 0.44h_2l_1$
$1^1B^+_{3u}$ (S_4)	6.74	1.5535	$0.47hl_1 + 0.46h_1l$
$1^1B^+_{1g}$ (S_5)	6.82	0.0000	$0.51hl_2 + 0.41h_2l$
$2^1B^+_{2u}$ (S_6)	6.93	0.3265	$0.63h_1l_1$
TDA/TDDFT/LRC-ωPBEh/def2-SVP			
$1^1B^-_{3u}$ (S_1)	4.74	0.0000	$0.70h_1l - 0.70hl_1$
$1^1B^+_{2u}$ (S_2)	5.02	0.0954	$0.93hl$
$1^1B^-_{1g}$ (S_3)	6.52	0.0000	$0.77hl_2 - 0.63h_2l$
$2^1A^-_g$ (S_4)	6.61	0.0000	$0.68h_1l_2 - 0.66h_2l_1$
$1^1B^+_{3u}$ (S_5)	6.77	2.0044	$0.68hl_1 + 0.68h_1l$
$2^1B^+_{2u}$ (S_6)	6.92	0.3631	$0.89h_1l_1$
MRSF-TDDFT/LRC-ωPBEh/def2-SVP			
$1^1B^-_{3u}$ (S_1)	4.34	0.0019	$0.71h_1l + 0.67hl_1$
$1^1B^+_{2u}$ (S_2)	4.63	0.4469	$0.99hl$
$1^1B^+_{3u}$ (S_3)	5.47	2.3264	$0.72hl_1 - 0.68h_1l$
$1^1B^-_{1g}$ (S_4)	5.89	0.0000	$0.68hl_2 - 0.69h_2l$
$1^1B^+_{1g}$ (S_5)	6.18	0.0000	$0.71hl_2 + 0.69h_2l$
$2^1B^+_{2u}$ (–)	Not available		

[a] Dominant configurations and coefficients; h_xl_y refers to the HOMO−x →LUMO+y transition.

2 Analysis of the electronic configurations generated by MRSF-TDDFT with different triplet references

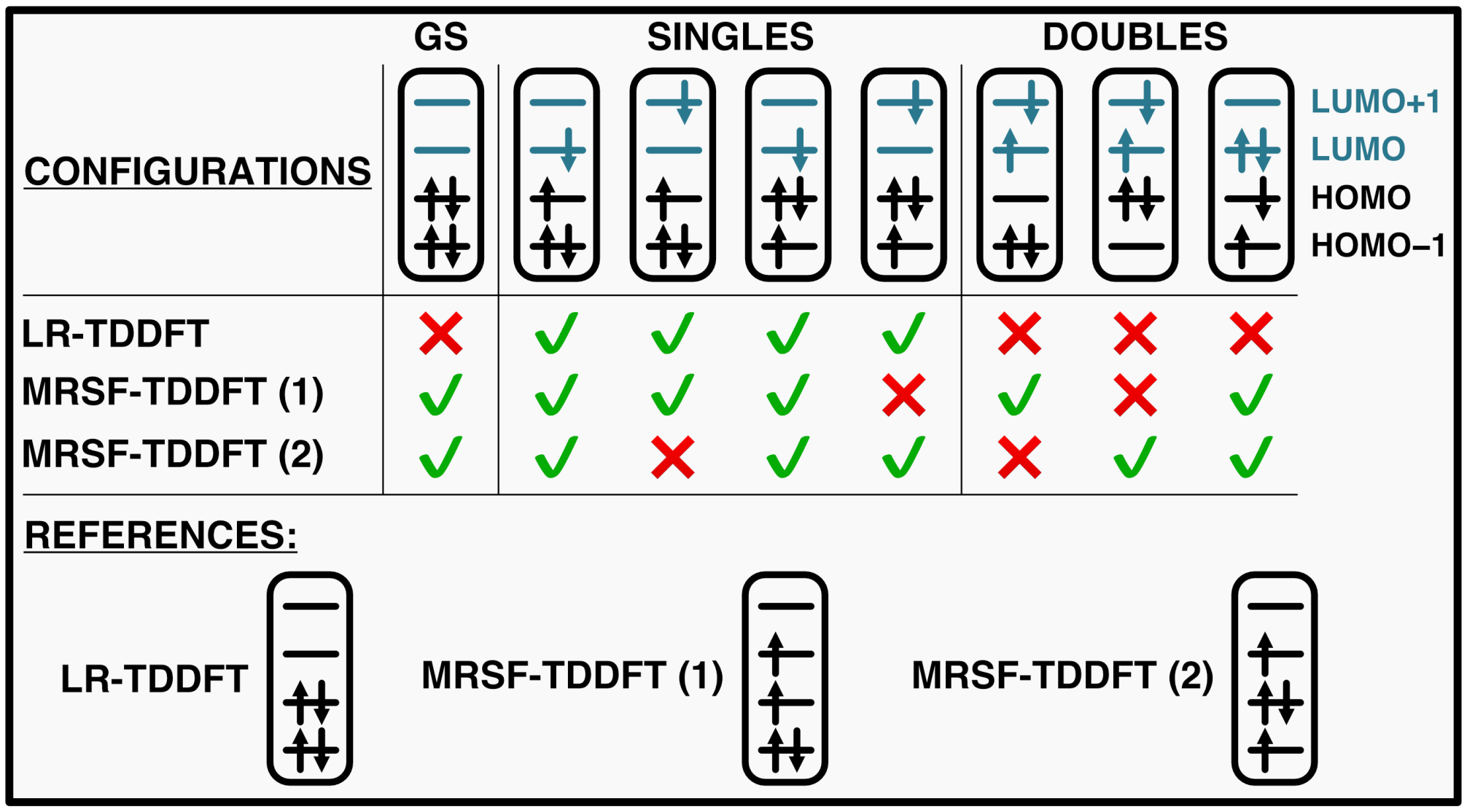

Figure S1: Simplified schematic of the electronic configurations generated by LR-TDDFT and MRSF-TDDFT from two references with different singly-occupied orbitals, as appearing in the case of *ortho*-nitrophenol. The schematic demonstrates that having different occupations of orbitals in the triplet reference of MRSF-TDDFT leads to not fully-overlapping sets of electronic configurations, which are then used to generate the response electronic states. Hence, the response electronic states generated from the two triplet references would not be the same in general. Note that extending the MRSF-TDDFT formalism to include the missing singly-excited configurations would enlarge the overlap between the sets, yet the doubly-excited configurations would still make a difference.

3 The effect of enforcing orbital continuity in MRSF-TDDFT: the case of ortho-nitrophenol

Enforcing orbital continuity, for example, by using the maximum overlap method (MOM), offers a viable strategy to avoid discontinuities caused by **Limitation 2**. However, enforcing orbital continuity carries several drawbacks. First, the enforced orbitals might not be representative of the electronic state characters in the nuclear configuration space explored by the trajectories. Then, enforcing a certain set of orbitals might bias the results. Second, one should decide which orbitals should be enforced along the trajectory, which requires expertise and benchmarking similar to selecting the active space for multiconfigurational methods. Hence, MOM would either have to be used blindly, or MRSF-TDDFT would lose its highly appreciated black-box character.

We tested enforcing orbital continuity for the *ortho*-nitrophenol, see Fig. S2. While the stability was improved in the vicinity of the T_1/T_2 degeneracy, the orbital continuity became difficult to enforce once the T_1 and T_2 states became too separated in energy. Moreover, the results highlight the strikingly different character of states for the two references. In such a case, it becomes unclear how to define the reference.

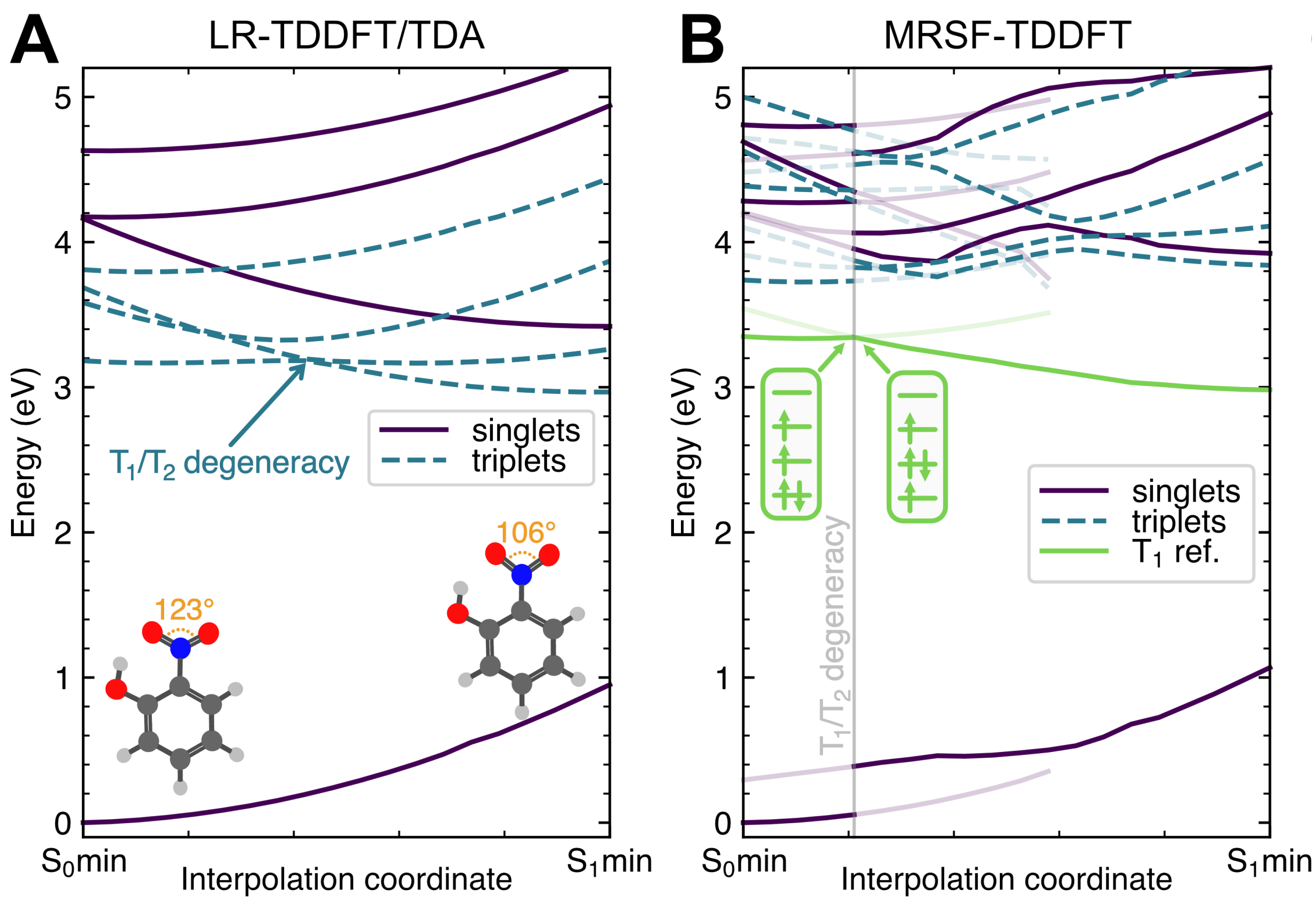

Figure S2: Electronic energies along a geodesic interpolation between minima on the S_0 and S_1 electronic states of *ortho*-nitrophenol. The two geometries, taken from Ref. S3, differ by a small alteration of the O–N–O angle. **A**: LR-TDDFT/TDA/ωB97X-D/def2-TZVP calculations. **B**: MRSF-TDDFT/ωB97X-D/def2-TZVP calculations. The plot contains the reference triplet state (solid green line) and the response triplet states (dashed blue lines). The T_1/T_2 point of degeneracy for the reference triplet state is marked by a grey vertical line. The electronic configurations of the reference at each side of the degeneracy are depicted as an inset. The shaded lines correspond to results with T_2 taken as a reference, enforced by orbital continuity. Note that the states from the T_1 and T_2 references are both qualitatively and quantitatively different.

4 Ethyl diazoacetate results with the CAM-B3LYP functional

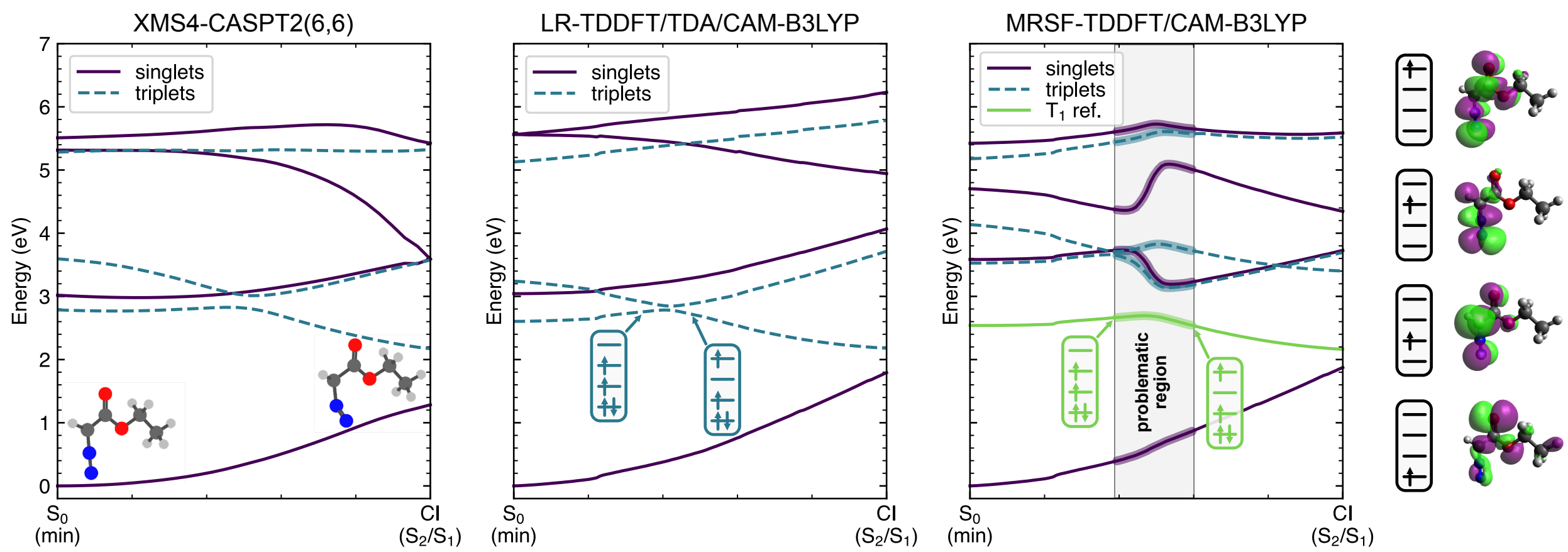

Figure S3: Electronic energies along a geodesic interpolation between the S_0 minimum and the S_2/S_1 MECI of ethyl diazoacetate, optimized at XMS4-CASPT2(6,6)/cc-pVDZ level of theory. **A**: XMS-CASPT2(6,6)/cc-pVDZ calculation. **B**: LR-TDDFT/TDA/CAM-B3lYP/cc-pVDZ calculation. **C**: MRSF-TDDFT/CAM-B3lYP/cc-pVDZ calculation. The plot contains the reference triplet state (solid green line) and the response triplet states (dashed blue lines) calculated with MRSF-TDDFT. The problematic region around the T_1/T_2 avoided crossing of the reference triplet state is marked by a grey rectangle. **D** Four frontier orbitals of ethyl diazoacetate.

5 Active-space orbitals used in XMS4-CASPT2(6,6)

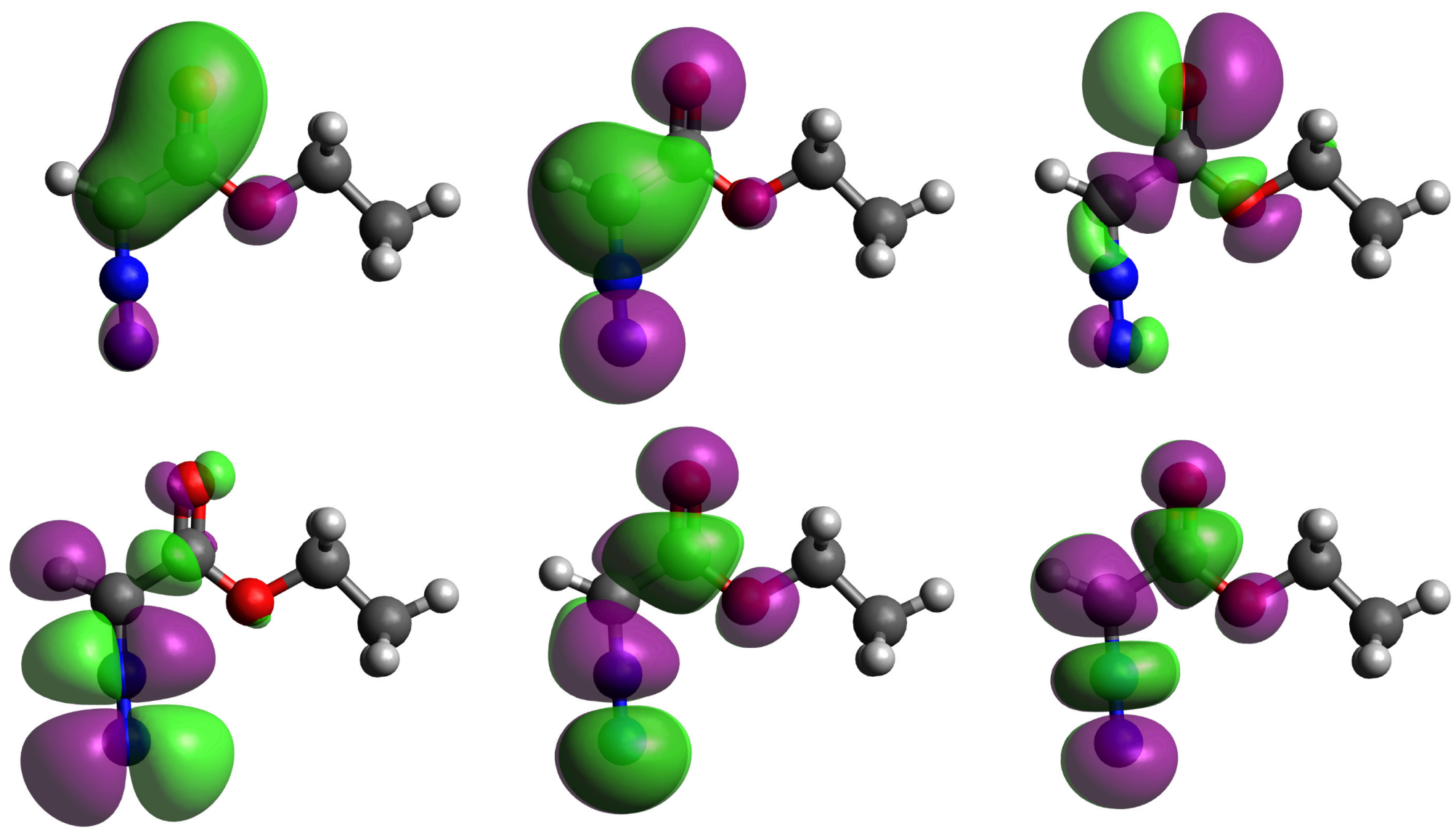

Figure S4: Orbitals composing the active space of the XMS4-CASPT2(6,6)/cc-pVDZ method used for ethyl diazoacetate.

References

(S1) Kimber, P.; Plasser, F. Energy Component Analysis for Electronically Excited States of Molecules: Why the Lowest Excited State Is Not Always the HOMO/LUMO Transition. *Journal of Chemical Theory and Computation* **2023**, *19*, 2340–2352.

(S2) Hashimoto, T.; Nakano, H.; Hirao, K. Theoretical study of the valence $\pi \rightarrow \pi^*$ excited states of polyacenes: Benzene and naphthalene. *The Journal of Chemical Physics* **1996**, *104*, 6244–6258.

(S3) Greene, H. J. M.; Ghosh, D.; Sazanovich, I. V.; Phelps, R.; Curchod, B. F. E.; Orr-Ewing, A. J. Competing Nonadiabatic Relaxation Pathways for Near-UV Excited ortho -Nitrophenol in Aqueous Solution. *The Journal of Physical Chemistry Letters* **2024**, *15*, 9153–9159.
